# The Compulsory Notification of Pulmonary Tuberculosis

**Published:** 1911-12-02

**Authors:** 


### THE COMPULSORY NOTIFICATION OF PULMONARY TUBERCULOSIS.

THERE can be little doubt that the medical profession as a whole will welcome the order issued by the Local Government Board making compulsory the notification of pulmonary tuberculosis. This order may be regarded as the logical outcome of the campaign which has been developing during the last ten years or so for the prevention and control of phthisis.

The first step was voluntary notification, which was adopted in England in 1899. This was followed by compulsory notification in a few of the larger provincial towns, notably in Sheffield, where a special Act to this effect was obtained in 1904; a year or two later Bolton and Edinburgh followed the example of Sheffield. In 1908 notification was made compulsory in the case of all Poor Law patients, and early in the present year this regulation was extended to all patients in any public institution in London. In America voluntary notification of phthisis was introduced in 1894, and three years later it was made compulsory.

### Objections to Notification.

The opposition to compulsory notification has been based on two main grounds. In the first place, it has been thought that such a system might lead to a very irksome interference with the privacy of the home and of the family, and would attach an unwarrantable stigma to the consumptive patient. In the second place, many eminent authorities consider that the results of voluntary notification have been disappointing, and that any form of notification is of little value unless it can be followed up by suitable remedial and curative measures, such as are, unfortunately, unattainable at present in the majority of cases.

The first of these objections is the more easy to meet, for past experience has shown that all information given to the local authority in the course of notification has been treated in the strictest confidence, and we are glad to see that the new order insists on the importance of maintaining these confidential relations. With regard to the second objection, it was originally hoped that under the voluntary system a large proportion of all known cases of pulmonary tuberculosis would be notified to the local authorities, who would thus be enabled to form a fairly accurate estimate of the amount and distribution of the disease in their respective areas, and could then take the proper steps to control it.

### Small Number Notified.

Unfortunately these results have not been obtained, and only a small percentage of cases has been notified. In a recent report on this subject to the London County Council, Dr. Wanklyn states that in the twenty-two London districts in which notification is voluntary, the number of cases of phthisis notified was actually *less* than the number of deaths from the disease. If we reckon the number of living cases of phthisis as five times the number of deaths in any one year—and this is a low estimate—it would appear that only about 12 per cent of all cases are notified.

These disappointing results are largely due to a widespread opinion among the medical profession that notification is likely to be of so little value to their patients that they are scarcely justified in putting them to the slight inconvenience which such a step entails.

### A Necessary Corollary.

The procedure followed by the local authority on receiving a notification varies in different districts, but speaking generally it is confined to inspection and, if necessary, disinfection of the patient's house, and to giving instructions as to personal hygiene and the avoidance of infecting other people. This is all to the good so far as it goes. But it must be clearly understood that no system of notification, whether voluntary or compulsory is likely to produce a satisfactory result unless it is followed up by a continuous observation of the patient, by the provision of suitable treatment at home or in a sanatorium, and by the careful examination of all "contacts" for the early signs of the disease. Such an extended form of supervision the local authorities have not as yet provided except in a few instances, and then only in a limited and tentative way.

### The Effect of Dispensaries.

There are, however, certain voluntary institutions —the so-called "Tuberculosis Dispensaries"— which are trying with considerable success to cope with the difficulty. The first of these was established in Edinburgh some twenty years ago, and others have recently been started in London and other large towns. These dispensaries are supported by voluntary enterprise, but work in close co-operation with the Medical Officer of Health of the district, to whom all their cases are notified. They are also in touch with the local branch of the Charity Organisation Society and other kindred societies, and in this way are usually able to obtain letters of admission to a sanatorium, or grants of money to improve the patient's home conditions, when these seem to require it. Great importance is attached to the careful examination of all "contacts," and in this way a number of very early cases are discovered, in which suitable treatment holds out a good chance of recovery.

It is to be hoped that this new order will stimulate the local authorities throughout the country to follow the lead given by these dispensaries. If they will but do so, not only will an enormous number of early cases of phthisis, of which we are at present entirely ignorant, be brought to light; but also a very great advance will have been made towards the stamping out of what is, after all, a preventible disease.

